# Associations Between Vocational Interests and Personality “Beyond” the Big-Five

**DOI:** 10.1177/00332941241311497

**Published:** 2025-01-06

**Authors:** Sereena Dargan, Nicole Carrier, Julie Aitken Schermer

**Affiliations:** Department of Psychology, 151157 The University of Western Ontario, London, ON, Canada; Departments of Psychology and Management and Organizational Studies, Faculty of Social Science, 151157 The University of Western Ontario, London, ON, Canada

**Keywords:** Vocational interests, career interests, personality, personality assessment

## Abstract

This investigation explores the relationships between vocational interests and personality dimensions suggested to be “beyond” the Big Five or Five Factor Model. Participants (653 adults; 125 men and 528 women, with a mean age of 40.57 years, *SD* = 16.61, range 18–92) provided data on the 10 personality dimensions of the Supernumerary Personality Inventory (SPI; Paunonen, 2002), along with the 34 scales (27 work interests and seven work style preference scales) of the Jackson Career Inventory. Several significant associations, such as negative correlations between conventionality and an interest in the arts, negative correlations between femininity and an interest in science, positive correlations between humorous and an interest in nature/adventure/medicine, and positive correlations between manipulativeness and an interest in business were identified bringing to light new perspectives on how personality relates to different occupational roles and styles. The findings stress the importance of employing diverse personality and vocational interest measures to provide a more holistic view of associations between personality and career preferences. Limitations and future research are discussed.

## Introduction

In today’s organizational climate, where careers demand significant investments of time and energy, many individuals seek career paths that align with their personal characteristics such as interests, values, personality, and aptitudes. Organizational research has focused particularly on how evaluating elements of personality and vocational interests might facilitate self-reflection and self-discovery, enabling individuals to make career decisions to which they are personally suited (Carless, 1999). To this end, several theories and assessments of vocational interests have been developed and are used in research and practice to relate such interests to existing educational and employment domains. Understanding how these interest domains relate to personality can enable individuals to make career decisions that are both satisfying and well-suited to their inherent characteristics. The current study adds to the existing personality and vocational interest literature by exploring the associations between the factors and scales of the Jackson Career Explorer (JCE; Schermer et al., 2012) and the Supernumerary Personality Inventory (SPI; Paunonen, 2002).

### Personality and Vocational Interests

Extensive research has furthered knowledge on how individuals with certain personality traits might display an affinity for vocations throughout their educations and careers. For instance, early studies by Darley (1938) and Berdie (1943) report comparisons of personalities in male students based on their occupational interest patterns. Students with lower social inclinations showed an interest in technical occupations such as engineering (Berdie, 1943; Darley, 1938). These students reported higher levels of masculinity, a characteristic shared with students who identified an interest in a skill-based trade (Berdie, 1949). Differently, higher social preferences and emotional stability were associated with an interest in social welfare or forward-facing business and sales-related occupations (Berdie, 1943; Darley, 1938).

Later investigations employed more formalized methods of assessment and categorization that allowed for easier replication, larger analyses, and comparison across studies. For instance, Holland’s (1997) model emerged as a common method of categorizing vocational interests, including six interest and work domains: realistic (preferring hands-on practical tasks), investigative (analytic and problem solving), artistic (creative and expressive), social (involving helping and interacting), enterprising (leading and persuading), and conventional (structured and task-based) (or RIASEC) and providing a framework for understanding career preferences. The RIASEC domains are most often examined with the widely used Five-Factor Model of personality (FFM; Digman, 1990) encompassing the broad trait dimensions of openness, conscientiousness, extraversion, agreeableness, and neuroticism. Review studies that have explored associations between these models (see Ackerman & Heggestad, 1997; Barrick et al., 2003; Larson et al., 2002; Tokar et al., 1998) generally report consensus on positive associations between trait extraversion and enterprising and social career domains (Ackerman & Heggestad, 1997; Barrick et al., 2003; Larson et al., 2002; Tokar et al., 1998) as well as between trait openness and artistic and investigative career domains (Larson et al., 2002; Tokar et al., 1998). Weaker positive correlations are also noted between traits of agreeableness and social career interests (Barrick et al., 2003; Larson et al., 2002; Tokar et al., 1998), as well as trait conscientiousness and conventional interests (Ackerman & Heggestad, 1997). The findings observed across studies represent significant progress in understanding personality-interest relations concerning vocational assessment and decision-making and have informed discussions on the distinction between these self-related concepts (Barrick et al., 2003; Holland, 1997) by drawing attention to the uniqueness and overlap in their assessment measures. Notably, the importance of extending these investigations to include other common and validated personality and interest models is also apparent. McKay and Tokar (2012) include the HEXACO personality model (Ashton & Lee, 2001), which outlines modified definitions and sub-features of FFM traits (particularly agreeableness and neuroticism) as well as the addition of trait honesty-humility. McKay and Tokar’s (2012) study reinforced some previous findings on links between trait openness and artistic and investigative career domains, as well as traits of agreeableness and extraversion with the social interest domain. Additionally, novel findings surrounding the trait honesty-humility were found, which showed significant positive associations with social career interests, and a negative association with enterprising interest (McKay & Tokar, 2012). By demonstrating the increased predictive validity of the HEXACO over the FFM in explaining variance in RIASEC dimensions, the results reported by McKay and Tokar (2012) demonstrate the potential in introducing new dimensions and perspectives in vocational assessment research.

### Extending Traditional Measures of Vocational Interest and Personality

#### The Jackson Career Explorer

While Holland’s (1997) RIASEC model remains prominent in organizational literature, other measures have been put forth that offer extended representations of vocational interest (Jackson, 1977, 2000). The present study focuses on the Jackson Career Explorer (JCE; Schermer et al., 2012), a modified version of the Jackson Vocational Interest Survey (JVIS; Jackson, 2000), which has gained popularity owing to its enhanced utility and practicality. The original JVIS includes 289 pairs of forced-choice behavioural statements reflecting activities typical of certain occupational fields. The items comprise 34 scales (27 work interests and seven work style preference scales) that assess individuals’ preferences for different occupational roles and styles. The JVIS also represents a strong assessment tool, however, it presents a few notable drawbacks such as the length, and the forced-choice response format that limits the scale’s utility in multivariate analyses. In developing the JCE, Schermer and Vernon (2008) reduced the length by including only the five items with the strongest item-scale correlations from each of the 34 scales of the JVIS. The forced-choice response format was also replaced with a Likert-type response scale in which individuals rate their predicted enjoyment of each activity. The 34 interest scales retained in the JCE can also be further categorized based on seven broader factors labelled artistic, science, social, biology, applied, business, and work Style (Schermer & Vernon, 2008). The properties and validity of the JCE have been demonstrated through comparisons with other vocational measures such as the Career Directions Inventory (Jackson, 2003) and RIASEC interest domains described in Holland’s (1985) Vocational Preference Inventory (Schermer, 2012; Schermer & MacDougall, 2011). For instance, Schermer (2012) notes high positive correlations between Holland’s (1985) realistic domain and JCE scales for engineering, nature-agriculture, and skilled trades. The investigative domain of Holland’s (1985) inventory was appropriately correlated with JCE scales for physical science, engineering, and life science. The artistic domain correlated most strongly with JCE scales for creative arts, performing arts, and author-journalism. The social domain demonstrated its highest correlations with JCE scales for social science, teaching, social service, and elementary education. The enterprising domain was strongly correlated with multiple JCE scales including personal service, finance, business, sales, supervision, human relations management, law, and professional advising. The final RIASEC domain, conventionality, correlated with JCE scales for finance, business, sales, supervision, human relations management, law, and professional advising (Schermer, 2012). In addition, the JCE appears not to be strongly impacted by social desirability responding, making it less susceptible to these impression management biases which affect many personality and self-report scales (Schermer, 2019).

In research assessing the associations between the JCE scale and personality traits, Schermer (2012) examined the correlations with the five personality factors (extraversion, intellect, agreeableness, conscientiousness, and neuroticism) derived from the 20 Adjective Rating Scale (Paunonen & Jackson, 1987) and found meaningful correlations between the two measures, resembling past findings from research employing other career interest assessments. Kowalski et al. (2017) demonstrated that several of the JCE interest factors and scales are associated with socially malevolent personality traits. For example, Kowalski et al. (2017) reported positive correlations between Machiavellianism and social interests, between sub-clinical psychopathy and scientific interests, and between sub-clinical narcissism and artistic interests. Velji et al. (2023) expanded on the research topic by investigating facets of narcissism with the JCE and found dimensions such as need for admiration positively correlating with an artistic interest and having a grand fantasy positively associated with an interest in business. The findings of these past studies have provided strong support for the validity and utility of the JCE, as well as a fuller understanding of the characteristics and profiles of individuals who generally endorse these career preferences.

#### The Supernumerary Personality Inventory

Although the FFM and HEXACO models provide solid frameworks for understanding and measuring personality, some researchers have turned attention toward additional personality traits that fall outside of the common factor space (Paunonen & Jackson, 2000). The Supernumerary Personality Inventory (SPI; Paunonen, 2002) was developed to measure traits that extend “beyond” those captured by traditional models. The SPI includes 10 scales, measuring the traits of conventionality, seductiveness, manipulativeness, thriftiness, humorousness, integrity, femininity, religiosity, risk-taking, and egotism. The SPI has been used alongside other measures to highlight associations between these less-examined traits and various characteristics and outcomes such as altruism, donation behaviour, pro-environmental values and behaviours, humour styles, materialism, counterproductive work behaviour, and unethical behaviours (Dargan & Schermer, 2022; Hong et al., 2012; Kfrerer & Schermer, 2020; Kowalski et al., 2021; O’Neill & Hastings, 2011; Simpson et al., 2021). However, how the personality traits captured by the SPI are associated with dimensions of vocational interest has yet to be explored. The present study is exploratory and aims to examine associations between personality dimensions of the SPI and vocational interest factors of the JCE. To do this, correlations between the scales will be examined, along with the effects of gender and age. Regression analyses will further address whether personality dimensions described by the SPI predict JCE interest factors.

## Method

### Participants and Procedure

Participants were a combination of 653 (125 men and 528 women) undergraduate management and organizational studies students and community volunteers. The average age was 40.57 years (*SD* = 16.61) and ranged from 18 to 92. There were no incentives to complete the study and participants provided informed consent prior to completing any of the survey items. The study was completed online using Qualtrics. Institutional ethical approval was granted.

### Instruments

Following completion of demographic items asking for the individual’s biological sex (sex assigned at birth) and age, participants completed the 150-item SPI (Paunonen, 2002), consisting of 15 items for each of the 10 personality scales, responded to using a true/false response key. The SPI scales include conventionality (e.g., “*I find it hard to change my personal habits.*”), seductiveness (e.g., “*I will try to sit closer to someone I find attractive.*”), manipulativeness (e.g., “*I often think up ways to get what I want from other people.*”), thriftiness (e.g., “*I use a budget to monitor my spending.*”), humorousness (e.g., “*My behavior makes other people laugh*.”), integrity (e.g., “*If I found a wallet, I would not be tempted to keep any money.*”), femininity (e.g., “*My friends consider me to be somewhat feminine.*”), religiosity (e.g., “*The teachings of my religion are sacred to me.*”), risk-taking (e.g., “*Sky-diving from an airplane appeals to me.*”), and egotism (e.g., “*I would rate myself as above average in most areas.*”). Coefficient alpha values range from .66 for conventionality to .95 for religiosity. Scale scores represent a linear aggregate, with higher scores representing a greater endorsement of the items for the scale.

Participants also completed JCE (Schermer et al., 2012), consisting of 170 items. Each item is presented individually and is responded to using a Likert scale from 1 *(Would not want to do this activity*) through to 5 (*Would very much like to do this activity*). The JCE measures 27 vocational interests (5 items per interest) that have been found to fit a six-factor solution including artistic, science, social, biology, applied, business, and a single work style factor consisting of seven style scales (Schermer & Vernon, 2008). The JCE has high internal consistency, with the average alpha for the scales being 0.76 (Schermer, 2012; Schermer & MacDougall, 2011; Schermer & Vernon, 2008).

## Results

Table 1 presents the correlations between the personality factors of the SPI and the interest scales and factors of the JCE. Significant correlations between personality traits and vocational interest factors ranged from small to large in effect (.102 ≤ *r* ≤ .523) and significant correlations between individual interest scales and personality were of similar magnitudes (.082 ≤ *r* ≤ .667). Table 2 presents the results of the regression analysis predicting JCE factor scores with age, sex, and SPI personality traits.

**Table 1.** Correlations Between JCE Vocational Interests and SPI Personality Traits.

	Con	Sed	Manip	Thrift	Humor	Integrity	Feminine	Relig	Risk	Egotism
*Artistic Factor*	−.204*	.207*	.138*	−184*	.250*	−.140	.050	−.019	.165*	.177*
Creative arts	−.131*	.138*	.057	−.173*	.038	−.076	.102*	−.045	.092	.116*
Performing arts	−.171*	.222*	.161*	−.151*	.287*	−.132*	.049	.032	.138*	.172
Author/Journalism	−.162*	.180*	.130*	−.156*	.289*	−.142*	.017	−.006	.126*	.145*
Technical writing	−.159*	.068	.059	−.036	.105*	−.051	−.030	−.077	.153*	.086
*Science Factor*	−.079	.212*	.226*	.045	.155*	−.097	−.327*	−.158*	.311*	.239*
Mathematics	−.046	.154*	.199*	.063	.071	−.015	−.232*	−.090	.149*	.240*
Physical science	−.059	.195*	.210*	.034	.137*	−.087	−.328*	−.139*	.300*	.228*
Engineering	−.003	.180*	.212*	−.009	.158*	−.154*	−.447*	−.114*	−.311*	.177*
Life science	−.156*	.174*	.124*	.045	.173*	−.082	−.107*	−.165*	.281*	.162*
*Social Factor*	−.027	−.004	−.006	−.011	−.065	.148*	.219*	.105*	.003	.167*
Social science	−.201*	.083	.066	.013	.149*	.010	.066	−.060	.153*	.176*
Personal service	.105*	.004	.031	−.068	.021	.075	.224*	.134*	−.059	.103*
Teaching	−.020	−.025	.030	.074	.049	.159*	.089	.052	−.030	.205*
Social service	−.061	.004	−.021	−.049	.059	.089	.206*	.116	.069	.079
Elementary ed	.043	−.039	−.082	.020	−.009	.188*	.229*	.141*	−.081	.075
*Biology Factor*	−.099	.208*	.131*	−.043	.258*	.134*	−.271*	−.048	.523*	.102*
Adventure	−.185*	.346*	.259*	−.120*	.288*	−.278*	−.385*	−.203*	.667*	.158*
Nature-agri	−.047	.079	.050	.036	.127*	−.063	−.165*	−.034	.280*	.004
Medical service	.008	.034	−.014	−.009	.140*	.032	−.059	.113*	.186*	.049
*Applied Factor*	.203*	−.130*	−.120*	.055	−.146*	.159*	.196*	.236*	−.233*	−.019
Skilled trades	.159*	.023	.052	.040	.041	−.091	−.206*	.094	.044	.033
Family activity	.044	−.071	−.095	−.070	−.107*	.194*	.333*	.150*	−.186*	.051
Office work	.232*	−.202*	−.171*	.134*	−.207*	.190*	.209*	.246*	−.288*	−.090
*Business Factor*	.061	.127*	.219*	−.049	.056	−.054	−.128*	−.034	.049	.310*
Dom. Leadership	.072	.119*	.237*	−.001	.087	−.067	−.123*	−.032	.088	.284*
Finance	.029	.161*	.187*	−.069	.008	−.113	−.180*	−.034	.105*	.210
Business	.072	.063	.152*	−.036	.011	.004	−.065	−.050	−.042	.252*
Sales	.102*	.080	.142*	−.050	.005	−.027	−.023	.066	−.049	.185*
Supervision	.028	.074	.153*	−.019	.042	−.018	−.098	−.044	.000	.311*
HR management	.013	.094	.162*	−.008	.092	−.022	−.101*	−.022	.068	.288*
Law	.058	.087	.130*	−.060	.035	−.060	−.146*	−.007	.098	.176*
Prof. Advising	−.016	.167*	.231*	−.095	.097	−.061	−.087	−.070	.085	.278*
*Work Style Factor*	.111*	−.079	−.139*	.125*	−.065	.262	.043	−.004	−.160*	.140
Job security	.219*	−.069	−.059	.118*	−.145*	.144*	.090	.085	−.214*	.007
Stamina	.139*	.130*	−.166*	.065	−.079	.238*	.059	.011	−.130*	.042
Accountability	.049	−.206*	−.278*	.076	−.145*	.371*	.144*	.082*	−.172*	−.078
Academic- ach	−.144*	.038	−.002	.059	.077	.114*	.031	−.074	.042	.163*
Independence	.013	.043	−.013	.049	.012	.036	−.119*	−.123*	.013	.207*
Planfullness	.199*	−.196*	−.227*	.175*	−.221*	.310*	.084	.070	−.297*	−.009
Interpersonal-confidence	−.016	.058	−.011	.013	.081	.138	.004	−.024	−.039	.249

*Note.* Con = Conventionality; Sed = Seductiveness; Manip = Manipulativeness; Thrift = Thriftiness; Humor = Humorousness; Relig = Religiosity; Risk = Risk-taking; Ed = Education; Agri = Agriculture; Dom = Dominant; Prof = Professional; Ach = Achievement *p < .001, two-tailed.

**Table 2.** Regression Weights for Predicting JCE Factor Scores With Age, Sex, and SPI Scale Scores.

	Predictors	Standardized beta	*t*	*R*^2^ (adjusted *R*^2^)	*F*
Artistic	Sex	.105	2.258	.149 (.133)	9.187*
	Age	−.029	−.661		
	Conventionality	−.166	−3.790*		
	Seductiveness	.060	1.098		
	Manipulativeness	−.033	−.635		
	Thriftiness	−.060	−1.449		
	Humorousness	.162	3.799*		
	Integrity	−.045	−.934		
	Femineity	.061	1.258		
	Religiosity	.120	2.812		
	Risk-taking	.063	1.237		
	Egotism	.121	2.666		
Science	Sex	−.180	−4.058*	.235 (.220)	16.176*
	Age	−.056	−1.338		
	Conventionality	−.009	−.206		
	Seductiveness	.040	.783		
	Manipulativeness	.080	1.618		
	Thriftiness	.091	2.316		
	Humorousness	.026	.638		
	Integrity	.171	3.753*		
	Femineity	−.152	−3.299*		
	Religiosity	−.027	−.657		
	Risk-taking	.182	3.759*		
	Egotism	.170	3.946*		
Social	Sex	.197	4.235*	.163 (.147)	10.197*
	Age	.024	.550		
	Conventionality	−.018	−.426		
	Seductiveness	−.042	−.790		
	Manipulativeness	−.011	−.217		
	Thriftiness	.030	.719		
	Humorousness	.084	1.999		
	Integrity	.149	3.119		
	Femineity	.153	3.141		
	Religiosity	.102	2.396		
	Risk-taking	.184	3.649*		
	Egotism	.232	5.143*		
Biology	Sex	.078	1.890	.337 (.324)	26.724*
	Age	−.104	−2.656		
	Conventionality	.121	3.151		
	Seductiveness	−.025	−.526		
	Manipulativeness	−.039	−.856		
	Thriftiness	.033	.906		
	Humorousness	.129	3.469*		
	Integrity	.103	2.431		
	Femineity	−.125	−2.900		
	Religiosity	.102	2.713		
	Risk-taking	.562	12.457*		
	Egotism	.042	1.049		
Applied	Sex	.251	5.406*	.154 (.138)	9.573*
	Age	−.037	−.832		
	Conventionality	.147	3.360*		
	Seductiveness	.032	.588		
	Manipulativeness	−.060	−1.157		
	Thriftiness	.027	.660		
	Humorousness	−.071	−1.679		
	Integrity	−.013	−.274		
	Femineity	−.014	−.287		
	Religiosity	.148	3.479*		
	Risk-taking	−.051	−.997		
	Egotism	.044	.966		
Business	Sex	−.013	−.270	.124 (.107)	7.302*
	Age	.075	1.652		
	Conventionality	.035	.775		
	Seductiveness	−.004	−.071		
	Manipulativeness	.123	2.306		
	Thriftiness	−.023	−.545		
	Humorousness	−.056	−1.284		
	Integrity	.081	1.656		
	Femineity	−.100	−1.989		
	Religiosity	.004	.096		
	Risk-taking	.032	.612		
	Egotism	.283	6.096*		
Work style	Sex	−.009	−.198	.145 (.128)	8.446*
	Age	−.016	−.348		
	Conventionality	.077	1.707		
	Seductiveness	.041	.743		
	Manipulativeness	−.174	−3.255*		
	Thriftiness	.050	1.159		
	Humorousness	.029	.665		
	Integrity	.253	5.106*		
	Femineity	−.054	−1.071		
	Religiosity	−.102	−2.339		
	Risk-taking	−.083	−1.598		
	Egotism	.252	5.431*		

*Note.* *p < .001; two-tailed; Sex is coded 1 = men, 2 = women.

The artistic career interest factor was significantly positively correlated with traits of seductiveness, manipulativeness, humour, risk-taking, and egotism (*r* = .138 – .250, *p* < .001) and negatively correlated with conventionality and thriftiness (*r* = −.204 and .184, *p* < .001). The strongest trait correlations were with conventionality, seductiveness, and humour. Of interest, the personality dimension, conventionality, showed significant negative correlations with all four interest scales of the artistic factor. The results of the regression analysis in Table 2 also identify conventionality as a significant negative and humorousness as a significant positive predictor of the overall factor.

The science factor exhibited small to moderate positive correlations with traits of seductiveness, manipulativeness, humour, risk-taking, and egotism (*r* = .155 – .311, *p* < .001) and negative correlations with femininity and religiousness (*r* = −.327 and − .158, *p* < .001). Apart from humour and religiousness, these traits were also significantly associated with all four of the science factor interest scales. However, risk-taking was correlated in the opposite (negative) direction with the engineering interest scale. Notably, one of the stronger correlations found in the study also appeared between the interest scale of engineering and trait femininity. Femininity remained significantly associated with the science factor when examined in the regression. Trait integrity, which showed only a small negative correlation with the interest scale of engineering, was a significant positive predictor of the JCE science factor alongside traits of risk-taking and egotism.

The social career interest factor was positively correlated with SPI traits of integrity, femininity, religiousness, and egotism (*r* = .105 – .219, *p* < .001). Associations with femininity appeared the most robust, particularly with social interest scales of personal service, social service, and elementary education. In the regression analysis, the social factor was significantly and positively predicted by SPI traits of risk-taking and egotism.

The overall biology factor correlated positively with traits of seductiveness, manipulativeness, humorous, integrity, risk-taking, and egoism (*r* = .102 — .523, *p* < .001), and negatively with femininity. Traits of humorousness and risk-taking showed the most robust associations, exhibiting significant correlations with all three interest scales, including the largest correlation noted in this study between the adventure interest scale and trait risk-taking. Variance within the biology factor was also significantly predicted by traits of humorousness and risk-taking, with the latter showing a standardized effect of relatively large magnitude.

The applied factor of the JCE correlated positively with traits of conventionality, integrity, femininity, and religiousness (*r* = .159 — .236, *p* < .001), and negatively with seductiveness, manipulativeness, humour, and risk-taking (*r* = −.120 — −.233, *p* < .001). Under the applied factor, the interest scale for skilled trades exhibited significant negative correlations with trait femininity while correlations with family activity and office work were significant and positive. The regression analysis found traits of conventionality and religiosity to be significant positive predictors of the applied factor.

The business factor correlated positively with SPI traits of seductiveness, manipulativeness, and egotism (*r* = .127 — .310, *p* < .001), and correlated negatively with trait femininity. The strongest of these correlations were noted with manipulativeness and egotism which also showed significant association with most of the factor’s interest scales. Trait egotism was the only significant predictor of the business factor within the regression analysis.

Finally, the work styles factor exhibited small but significant positive correlations with trait conventionality and thriftiness along with negative correlations with manipulativeness and risk-taking. While the correlation between integrity and the overall factor did not reach significance, several of the correlations between integrity and the work styles interest scales (for instance, stamina, accountability, and planfulness) were moderate and significant. Within the regression, manipulativeness was a significant negative predictor, while traits of integrity and egotism positively predicted variance in the work styles factor.

### Sex Differences

Tables 3 and 4 display the results of the independent t-tests of sex difference and correlations with age for the JCE and SPI measures respectively. Significant sex differences were noted within several JCE factors and interest scales. Some of the most notable differences were found within the science, social, biology, and applied skills factors, which displayed sex differences in several of their interest scales. The science factor showed differences in mathematics, physical science, and engineering, with significantly higher means in men compared to women. In the regression analysis, reporting sex as a male was also a significant positive predictor of the science factor. In addition to the overall social factor, interest scales of personal service, social service, and elementary education showed significant sex differences in the direction of women. Results of the regression analysis agreed with these findings, revealing sex as a significant predictor of the social factor in the direction of women. The means found for the biology factor and its interest scale of adventure were significantly higher for men compared to women. Sex differences in the applied factor were attributed to differences in family activity and office work interest scales, for which higher means were reported by women. The effects of sex were also evident in the regression analysis which revealed reporting a gender of woman to be a significant positive predictor of this factor. A significantly higher mean was also found for men compared to women on the finance interest scale within the business factor. Rather, significantly higher means were found for women compared to men on interest scales of creative arts and the work style interest in accountability.

**Table 3.** Descriptive Statistics, Sex Difference Tests, and Correlations With Age for the JCE Factors and Scale.

	Men mean (SD)	Women mean (SD)	*t*	*r* with age
*Artistic Factor*	43.72 (16.58)	48.37 (16.20)	−2.86	−.17*
Creative arts	11.59 (4.70)	14.94 (5.65)	−6.11*	−.24*
Performing arts	10.27 (5.21)	11.21 (5.64)	−1.69	−.08
Author/Journalism	12.19 (6.10)	12.63 (6.03)	−.73	−.09
Technical writing	9.67 (4.20)	9.52 (4.30)	.35	−.11*
*Science Factor*	45.76 (19.33)	33.23 (13.79)	8.34*	−.17*
Mathematics	11.46 (6.17)	8.26 (4.75)	6.34*	−.12*
Physical science	12.10 (6.08)	7.78 (4.17)	9.39*	−.14*
Engineering	11.21 (5.70)	6.87 (3.20)	11.43*	−.06
Life science	11.08 (5.50)	10.37 (4.35)	1.54	−.21*
*Social Factor*	69.22 (18.04)	82.23 (19.29)	−6.79*	−.06
Social science	14.80 (5.28)	15.01 (5.19)	−.41	−.12*
Personal service	11.48 (4.01)	14.95 (4.48)	−7.89*	−.01
Teaching	16.69 (4.59)	18.11 (5.04)	−2.87	−.07
Social service	11.93 (4.85)	15.68 (5.53)	−6.96*	−.08
Elementary ed	14.54 (5.36)	18.21 (5.32)	−6.89*	.02
*Biology Factor*	38.39 (11.51)	34.54 (11.84)	3.27*	−.24*
Adventure	16.45 (5.28)	12.88 (5.29)	6.77*	−.35*
Nature-agri	11.65 (4.80)	10.96 (4.67)	1.48	−.12*
Medical service	10.28 (5.39)	10.70 (6.38)	−.68	.05
*Applied Factor*	32.85 (8.26)	39.19 (8.67)	−7.33*	.06
Skilled trades	8.84 (3.64)	7.90 (3.04)	2.67	−.01
Family activity	15.54 (3.89)	19.63 (3.66)	−11.06*	.01
Office work	8.55 (3.96)	11.52 (5.11)	−6.02*	.10
*Business Factor*	95.10 (30.30)	88.20 (28.33)	2.34	−.02
Dom. Leadership	13.76 (4.87)	12.31 (4.58)	3.11	−.12*
Finance	10.77 (5.51)	8.80 (4.51)	4.18*	−.08
Business	11.24 (4.38)	10.72 (4.22)	1.22	.03
Sales	7.86 (3.11)	7.96 (3.39)	−.29	−.05
Supervision	13.64 (4.70)	13.06 (4.74)	1.22	−.01
HR management	14.86 (5.08)	13.89 (4.92)	1.94	−.01
Law	11.13 (4.59)	9.87 (4.56)	2.75	.05
Prof. Advising	12.16 (4.70)	11.22 (4.57)	2.05	−.01
*Work Style Factor*	141.34 (15.61)	142.78 (16.56)	−.85	.04
Job security	17.60 (3.67)	18.15 (3.85)	−1.44	−.06
Stamina	20.96 (2.81)	21.19 (3.06)	−.75	.11*
Accountability	22.56 (2.46)	23.30 (2.20)	−3.26*	.11*
Academic- ach	19.03 (3.63)	19.21 (4.00)	−.44	−.06
Independence	19.31 (3.46)	18.29 (3.94)	2.65	−.01
Planfullness	21.01 (3.37)	21.60 (3.30)	−1.77	.16*
Interpersonal-confidence	20.30 (3.07)	20.96 (3.00)	−2.14	−.03

*Note.* Con = Conventionality; Sed = Seductiveness; Manip = Manipulativeness; Thrift = Thriftiness; Humor = Humorousness; Relig = Religiosity; Risk = Risk-taking; Ed = Education; Agri = Agriculture; Dom = Dominant; Prof = Professional; Ach = Achievement; *p < .001; two-tailed.

Regarding the SPI measure, Table 4 shows significantly higher means for men compared to women on traits of seductiveness, manipulativeness, humorousness, and risk-taking. Traits of integrity and femininity were reported as significantly higher in women compared to men.

**Table 4.** Descriptive Statistics, Sex Difference Tests, and Correlations With Age for the SPI Scales.

	Men mean (SD)	Women mean (SD)	*t*	*r* with age
Conventionality	51.90 (6.95)	50.99 (6.43)	1.39	30*
Seductiveness	43.49 (9.25)	40.08 (9.77)	3.54*	−.40*
Manipulativeness	42.72 (7.84)	39.63 (6.85)	4.39*	−.33*
Thriftiness	48.60 (8.02)	47.08 (7.64)	1.98	.12*
Humorousness	49.48 (8.40)	46.45 (9.15)	3.37*	−.14*
Integrity	55.86 (8.94)	60.63 (7.73)	−5.99*	.24*
Femininity	41.95 (8.47)	53.77 (6.06)	−17.99*	.02
Religiosity	43.78 (16.59)	48.82 (16.08)	−3.12	.28*
Risk-taking	45.15 (10.13)	38.13 (9.27)	7.45*	−.38*
Egotism	50.68 (6.90)	50.15 (6.71)	.78	−.29*

*Note.* *p < .001; two-tailed.

### Age differences

Correlation coefficients for age with JCE and SPI scales are in Tables 3 and 4 respectively. Significant correlations with age were noted for the JCE artistic, science, and biology factors. Specifically, the artistic factor correlated negatively with age, along with its interest scales of creative arts and technical writing. The significant negative correlation between age and the science factor was reflected in its interest scales of mathematics, physical science, and life sciences. The association between the biology factor and age were also negative, and significant negative correlations were found in interest scales of adventure and nature-agriculture.

Significant negative correlations were additionally found for the interest scales of social science and dominant leadership. Significant positive correlations with age were noted in the work style interest scales of stamina, accountability, and planfulness. To further examine the influence of age on career interest factors and scales, mean differences between individuals who were at or above typical retirement age (i.e., 65 or over; *n* = 63–73) versus those who were younger than 65 (*n* = 563–600) were explored. Independent groups *t* test results revealed several significant differences. Younger individuals scored significantly higher on the artistic factor *M*(*SD*) = 48.85 (16.54) versus 37.97 (12.95), *t* (659) = 5.35, *p* < .001 and its interest scale for creative arts *M*(*SD*) = 10.67 (5.14) versus 14.77 (5.59), *t* (667) = 5.93, *p* < .001, author/journalism *M*(*SD*) = 13 (6.15) versus 9.92 (5.03), *t* (668) = 4.11, *p* < .001, and technical writing *M*(*SD*) = 9.87 (4.39) versus 7.7 (3.05), *t* (112.26) = 5.42, *p* < .001. Younger individuals also scored higher on the biology factor *M* (*SD*) = 36.05 (11.79) versus 28.28 (9.98), *t* (659) = 5.33, *p* < .001 and its interest scale of adventure *M* (*SD*) = 13.96 (5.42) versus 9.82 (4.59), *t* (665) = 6.22, *p* < .001. Additionally, younger individuals scored significantly higher on interest scales for life science *M* (*SD*) = 10.79 (4.62) versus 8.36 (3.55), *t* (667) = 4.31, *p* < .001 and social service *M* (*SD*) = 15.28 (5.43) versus 12.58 (6.37), *t* (666) = 3.90, *p* < .001. When age was examined as a predictor within regression analysis, no significant associations with any of the JCE interest factors were found (see Table 2).

## Discussion

The goal of this study was to add to the current body of research on personality and vocational interests as well as to extend the validity and utility of the SPI and JCE. Results revealed several novel and significant correlations between the factors and scales of the two measures. Effect sizes ranged from small to large (Gignac & Szodorai, 2016), with many of the effect sizes being in the small to moderate range. Some notable findings included associations between the JCE science factor, particularly the engineering interest scale, and trait femininity (negative) as well as between an interest in adventure and risk-taking. The former aligns with current understandings of STEM fields as being male dominated. For example, only 13.9% of licensed engineers in Canada are women (Engineers Canada, 2020). While individuals higher in masculinity (i.e., lower in femininity) may generally show higher levels of interest in these vocations, societal biases and stigmatized perceptions of masculine and feminine roles may also be playing a role in the magnitude of this association. The latter finding is also intuitive given conceptualizations of adventurousness as entailing a willingness to approach situations that involve more novelty, risk, and unknown factors. Supporting this, risk-taking emerges as the strongest predictor of the biology factor which encompasses an interest in adventure.

The analysis of sex differences further highlights how interests and personality may be aligned with and/or influenced by traditional gender roles and expectations. Particularly, the higher scores for women in creative and social fields, along with higher scores in femininity and lower scores in risk-taking traits. Similarly, men’s higher scores in science and engineering point to a continuation of male dominance in STEM fields.

While the regressions predicting JCE factors and scales with age were non-significant, several significant findings related to age and the JCE were identified. Notably, the artistic and biology factors and interest scales for creative arts, technical writing, life sciences, and adventure were significantly associated with age through correlations and *t*-tests (comparing those younger vs. older than age 65), with younger individuals scoring higher on these dimensions. These findings suggest that career interests in some domains might decrease with age. However, attention should be drawn to the small subsample representing individuals over the age of 65 and the cross-sectional nature of the current study. Future investigations should incorporate samples with more balanced representations of ages to understand the impact of different lifespan stages on JCE career interest domains. In addition, longitudinal analyses would explain if vocational interests do decrease with age or if the results found here reflect generational differences. Other factors and interest scales also showed significant correlations with age, or mean differences between age groups. Although most reflected a lowering of interest with age, some positive correlations were noted, particularly within the interest scales of the work style factor. Additional results surrounding the negative associations between age and risk-taking, egotistic, and manipulative tendencies and the positive correlations with traits such as conventionality and integrity might suggest that older individuals attribute more value to traits involving stability, responsibility, and social conformity. It is possible that as individuals age, they prioritize careers and roles that align more with a sense of duty, reliability, and ethical conduct, reflecting a shift toward more socially valued and less risk-oriented personality traits and interests.

Given that the SPI was developed to capture traits “beyond” the traditional FFM dimensions, including this measure revealed associations that would have been overlooked in previous career literature. For example, the significant correlations between artistic interests and traits like seductiveness and humour suggest that creative career paths might be better understood through these additional dimensions. The presence of significant correlations also indicates the potential for increased predictive utility of personality assessments in vocational contexts. Further analyses of career interests using multiple models of personality are required to get a full understanding of the unique predictive ability of the SPI. Similarly, the JCE offers a more detailed breakdown of vocational interests compared to models such as the RIASEC, including 34 scales that assess specific preferences and interests represented by seven broader categories. Used together, these measures allow for a finer-grained analysis of how personality traits might align with distinct career paths. The results also offer practical implications as a more nuanced understanding of personality and interests can enhance self- and counsellor-facilitated vocational guidance and assessment.

Limitations of the current study include the sample diversity, particularly the imbalance of gender within the sample, the cross-sectional design, and the self-report nature of the included measures. Future research might aim to include more diverse samples in terms of gender and report on demographic variables such as social economic status and ethnicity to enhance the generalizability of findings. The cross-sectional nature also limits the ability to draw causal inferences. Further longitudinal investigations are required to better understand how personality and vocational interests develop and interact over time. Finally, incorporating other methods of data collection, such as behavioural assessments, supervisor or peer reports, as well as additional variables such as ability or job market trends are suggested to further validate and refine current findings on personality-interest relations. Overall, this represents one of the first (to our knowledge) investigations of correlations between SPI traits and JCE interest scales. The research contributes a more detailed understanding of the interplay between personality and preferences for certain vocational roles and styles and underscores the potential benefits of employing more refined and expansive measures in vocational interest research.

## Data Availability

The dataset analyzed for the current study is available in the Open Science Framework repository, https://osf.io/97u5f/?view_only=8611750fafda4bbf89e7c0671754f553.
